# Evaluation of Temporomandibular Joint Morphology According to the Occlusal Relationship Between Dental Arches Using Cone-Beam Computed Tomography

**DOI:** 10.3390/diagnostics16121784

**Published:** 2026-06-10

**Authors:** Busra Nur Gokkurt Yilmaz, Zerrin Unal Erzurumlu, Peruze Celenk, Suleyman Kutalmış Buyuk, Yeliz Kasko Arici

**Affiliations:** 1 Department of Dentomaxillofacial Radiology, Faculty of Dentistry, Giresun University, 28200 Giresun, Turkey; 2 Department of Dentomaxillofacial Radiology, Faculty of Dentistry, Ordu University, 52200 Ordu, Turkey; 3 Department of Dentomaxillofacial Radiology, Faculty of Dentistry, Ondokuz Mayıs University, 55270 Samsun, Turkey; 4 Department of Orthodontics, Faculty of Dentistry, Ordu University, 52200 Ordu, Turkey; 5 Department of Dental Biomaterials Science, Dental Research Institute, Seoul National University, Seoul 03080, Republic of Korea; 6 Department of Biostatistics and Medical Informatics, Faculty of Medicine, Ordu University, 52200 Ordu, Turkey

**Keywords:** angle classification, CBCT, morphology, temporomandibular joint

## Abstract

**Background**: This study aims to evaluate the temporomandibular joint (TMJ) morphology according to the occlusal relationship of the upper and lower dental arches shown on cone-beam computed tomography (CBCT) images. **Methods**: A total of 131 patients were evaluated using CBCT images and categorized as Angle Class I (Cl I), Class II (Cl II), or Class III (Cl III), based on the occlusal relationship of the dental arches. Measurements included the height and inclination of the articular eminence and the width and depth of the glenoid fossa for the right and left sides, as well as the angle between the long axis of both condyles and the angle between the long axis of the condyle and the midsagittal plane. A significance level of 5% was considered for all statistical analysis. **Results**: The articular eminence inclination and glenoid fossa depth demonstrated significant gender-related differences, while significant side-related variations were observed for articular eminence inclination, glenoid fossa width, and depth (*p* < 0.05). The articular eminence height was significantly higher in the Cl II (6.93 ± 1.07 mm) than in the Cl I (6.27 ± 1.22 mm) (*p* < 0.05) groups. The articular eminence inclination (best-fit line/top-roof line methods) also differed significantly among groups (*p* ≤ 0.001), with the highest values in the Cl II (51.74 ± 5.77°/38.27 ± 5.17°), followed by the Cl I (48.54 ± 5.94°/35.83 ± 4.43°) and Cl III (47.30 ± 7.36°/34.07 ± 5.24°) groups. No statistically significant differences were found among the study groups for glenoid fossa depth or width (*p* > 0.05). **Conclusions**: These findings suggest that TMJ morphology varies, depending on the occlusal relationship of the dental arches, gender, and side. These variations should be considered in both physiological and pathological evaluations of TMJ anatomy.

## 1. Introduction

The temporomandibular joint (TMJ) is a unique joint in the human body, regulating complex movements across multiple orthogonal planes and axes of rotation [1]. The TMJ is the only joint directly influenced by dental occlusion [2]. Occlusion exerts a significant influence on the functional position of the teeth, masticatory muscles, and the condyle within the TMJ, thereby contributing to the coordinated and balanced functioning of these structures [3]. Alterations in occlusal relationships may affect the distribution of mechanical loading on the TMJ surfaces, leading to variations in the joint’s adaptive response and resulting in morphological changes in the condylar structure. In turn, changes in condylar morphological adaptation may also influence occlusion [4].

Factors that may influence the morphological structure of the TMJ include age, gender, occlusion and malocclusion, TMJ diseases, TMJ disorders, emotional factors, and parafunctional habits [5,6]. The most significant morphological changes and positional asymmetries in TMJ structures are associated with tooth loss, dental wear, premature occlusal contacts, functional mandibular deviations, unilateral posterior crossbites, and dentoskeletal asymmetries. However, it is not yet known whether a specific morphological condition or joint position is typical for a particular type of malocclusion [7].

The TMJ can be imaged using conventional radiographs, panoramic radiography with TMJ programs, ultrasonography (US), cone-beam computed tomography (CBCT), computed tomography (CT), and magnetic resonance imaging (MRI) [8]. Three-dimensional imaging modalities allow for a more detailed evaluation of the TMJ [9]. The ability to obtain high-resolution images without superimposition in all three planes, along with relatively low radiation exposure and cost, are among the reasons why CBCT is preferred for evaluating TMJ bone changes [10].

A more comprehensive understanding of TMJ morphology in the context of the occlusal relationship between the dental arches may contribute to the physiological and pathological assessment of the TMJ. This study aims to evaluate the TMJ morphology according to the occlusal relationship of the upper and lower dental arches as seen on CBCT images. The null hypothesis of this study is that there is no association between the occlusal relationship of the upper and lower dental arches and TMJ morphology.

## 2. Materials and Methods

The present study was approved by the Ordu University Clinical Research Ethics Committee (approval date: 8 December 2023; approval number: 2023/325), and was conducted in compliance with the Declaration of Helsinki.

### 2.1. Study Design, Data Collection and Grouping

CBCT images obtained between 1 July 2020 and 20 August 2023 at the Department of Dentomaxillofacial Radiology Faculty of Ordu University were retrospectively evaluated. The CBCT images included in this retrospective study consisted of archived images that had been previously obtained for various clinical indications requiring three-dimensional radiographic evaluation of the dentomaxillofacial region. All images were acquired using KaVo OP 3D Vision (Imaging Sciences International LLC, Hatfield, PA, USA), and image analysis was conducted using OnDemand3D software (v.1.0, Cybermed Inc., Daejeon, Republic of Korea).

The determination of the sample size was performed using statistical power analysis with G*Power software version 3.1.9.7. A three-way ANOVA was utilized to compare the study groups (three groups) during the power analysis. Considering the medium effect size (d = 0.25) recommended by Cohen (1988) [11], the required minimum sample size was determined as 128 to achieve an 80% power at a 95% confidence level (α = 0.05).

The inclusion criteria comprised patients aged over 18 years, with images encompassing the upper and lower dental arches, both orbital bases, the porion point, and the TMJ region. CBCT images were excluded if they belonged to individuals with missing teeth (excluding third molars); asymmetric or cross-molar occlusion relationships; presence of cysts, tumors, or other pathologies in the TMJ region; a history of trauma or surgical procedures; orthodontic treatment (either ongoing or completed); or craniofacial anomalies. Additionally, CBCT images captured in an open-mouth position, without full occlusion of the teeth, or those with inadequate image quality were omitted from the present study.

Study groups were classified according to Angle’s dental classification based on the occlusal relationship of the dental arches observed on CBCT images as Class I (Cl I), Class II (Cl II), and Class III (Cl III) [12].

### 2.2. Standardization of CBCT Images and Measurements

The CBCT images included in the study were obtained at 120 kVp and 5 mA, with a maximum voxel size of 0.3 mm and a minimum field of view (FOV) of 16 × 6 cm.

**Figure 1:**
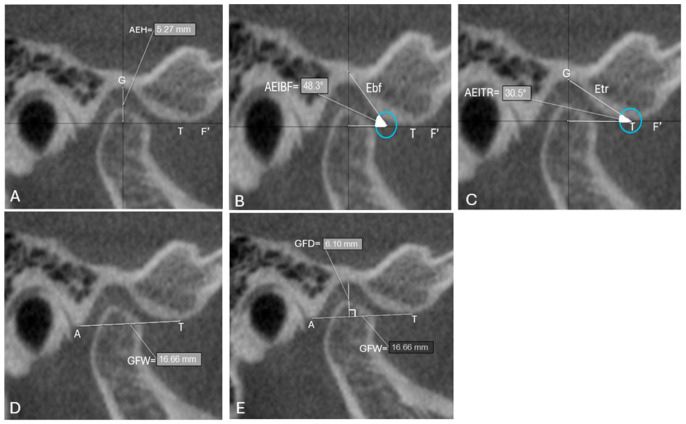
(**A**) Measurement of articular eminence height, (**B**) measurement of articular eminence inclination with the best-fit line method, (**C**) measurement of articular eminence inclination with the top-roof line method, (**D**) measurement of glenoid fossa width, and (**E**) measurement of glenoid fossa depth (G: the deepest point of glenoid fossa, T: the highest point of articular eminence, F’: line parallel to the Frankfurt horizontal plane passing through point T, AEH: height of articular eminence, Ebf: line tangent to the posterior inclination of articular eminence, AEIBF: articular eminence inclination according to the best-fit line method, Etr: line connecting point G and point T, AEITR: articular eminence inclination according to the top-roof line method, A: posterior point of glenoid fossa, GFW: width of glenoid fossa, and GFD: depth of glenoid fossa).

**Figure 2:**
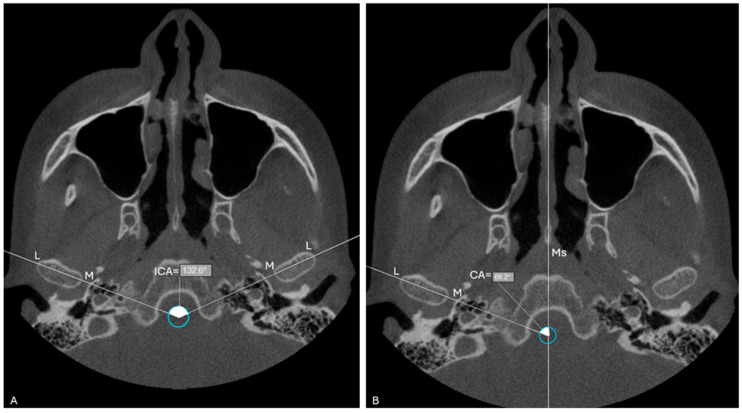
(**A**) Intercondylar angle measurement; (**B**) condylar angle measurement. (M: medial pole of mandibular condyle, L: lateral pole of mandibular condyle, ICA: intercondylar angle, Ms: midsagittal plane, and CA: condylar angle).

The articular eminence height (AEH) was measured as the vertical distance between the deepest point of the glenoid fossa and the highest point of the articular eminence. Its inclination was measured using two methods: the articular eminence inclination best-fit line (AEIBF) method, as the angle between a tangent to the posterior slope of the eminence and the FHP, and the top-roof line (AEITR) method, in which the angle was determined between a line passing through the highest point of the articular eminence and the deepest point of the glenoid fossa relative to the FHP. The glenoid fossa width (GFW) was measured as the distance from the highest point of the articular eminence to the posterior point of the glenoid fossa. The glenoid fossa depth (GFD) was measured as the perpendicular distance between the deepest point of the glenoid fossa and the line representing the GFW (Figure 1). The intercondylar angle (ICA) was determined by measuring the angle formed between the long axis of the right and left condyles. The condylar angle (CA) was defined as the angle between the long axis of each condyle and the midsagittal plane, and measurements were performed on both sides (Figure 2) [13].

All morphometric measurements were performed by an examiner who was blinded to the patients’ Angle classification groups. To assess intra-examiner reliability, all measurements were repeated by the same examiner after a 1-month interval. Measurements were repeated if differences exceeded 1 mm in linear measurements or 1° in angular measurements. The average of the two closest values was used for analysis.

### 2.3. Statistical Analysis

Data normality was evaluated using the Kolmogorov–Smirnov test, and homogeneity was evaluated with Levene’s test. Intraclass correlation coefficients (ICCs) with 95% confidence intervals were calculated to assess intra-examiner agreement between Measurement 1 and Measurement 2. A two-way ANOVA was used to analyze the intercondylar angle, whereas a three-way repeated-measures ANOVA was used for the remaining study variables, with side (right/left) treated as the within-subject repeated factor and gender and occlusal group as between-subject factors. Multiple comparisons were performed using the Bonferroni adjustment. Spearman’s rank correlation coefficient was calculated to examine the relationships between age and the study variables. To complement significance testing, effect sizes and precision estimates were reported. Partial eta squared (ηp^2^) was used as the ANOVA effect-size estimate, and mean differences with 95% confidence intervals and Cohen’s d values were calculated for significant Bonferroni-adjusted pairwise comparisons. All tests were two-tailed, and *p* < 0.05 was considered statistically significant. All statistical analyses were conducted using SPSS v28 software (IBM, Armonk, NY, USA).

## 3. Results

Of the 131 patients whose CBCT images were evaluated, 73.3% (*n* = 96) were female, and 26.7% (*n* = 35) were male. Based on the occlusal relationship of the dental arches, 38.2% (*n* = 50) of the patients had an Angle Cl I occlusion, 49.6% (*n* = 65) had an Angle Cl II occlusion, and 12.2% (*n* = 16) had an Angle Cl III occlusion. The patients mean age was 25.06 ± 7.99 years, with an age range of 18–52 years.

Although the main effects of side and gender on the AEH were not found to be statistically significant (*p* = 0.387; *p* = 0.078, respectively), a statistically significant difference was observed among the groups (*p* = 0.014). The mean AEH values were 6.27  ± 1.22 mm in Angle Cl I, 6.93  ± 1.07 mm in Angle Cl II, and 6.40  ± 1.48 mm in Angle Cl III. The mean AEH of patients with an Angle Cl II occlusion was significantly higher than that of those with an Angle Cl I occlusion (*p* < 0.05) (Table 1).

The main effects of the factors on the AEIBF were found to be statistically significant, with the mean AEIBF varying according to side (*p* = 0.001), gender (*p* = 0.012), and group (*p* = 0.001) (Table 2). Similarly, the main effects of the factors on the AEITR were also statistically significant, with the mean AEITR differing by side (*p* = 0.001), gender (*p* = 0.010), and group (*p* < 0.001) (Table 3).

Differences in the mean GFW were not statistically significant based on gender and groups (*p* = 0.316; *p* = 0.721, respectively), but significant differences were determined based on side (*p* = 0.029) (Table 4). The mean GFD did not show significant variation among groups (*p* = 0.052); however, it exhibited statistically significant differences based on side and gender (*p* = 0.004; *p* = 0.004, respectively) (Table 5).

The two-way ANOVA analysis showed that the mean ICA did not differ significantly according to gender or study group (Cl I, Cl II, and Cl III) (*p* > 0.05) (Table 6). Similarly, the three-way ANOVA results showed that the mean CA did not significantly vary according to side, gender, or study group (Cl I, Cl II, Cl III) (*p* > 0.05) (Table 7).

Partial eta squared (ηp^2^) values for the main effects of ANOVA are presented in Table 1, Table 2, Table 3, Table 4, Table 5, Table 6 and Table 7. Mean differences, 95% confidence intervals, and Cohen’s d values for statistically significant Bonferroni-adjusted pairwise comparisons are summarized in Table 8.

No significant statistical correlation was observed between age and the study variables (*p* > 0.05) (Table 9).

Intraclass correlation coefficients (ICCs) were determined to assess the agreement between measurements. The ICC values ranged from 88.0% to 99.99%, demonstrating strong agreement across all variables (*p* < 0.001). Specifically, the ICC for ICA was 0.999; for right and left CA, it was 0.998 and 0.999, respectively; for right and left AEH, 0.944 and 0.951; for right and left AEIBF, 0.995 and 0.996; for right and left AEITR, 0.993 and 0.993; for right and left GFW, 0.895 and 0.922; and for right and left GFD, 0.920 and 0.880.

According to the Bonferroni test, the difference between means with no common letter is significant (*p* < 0.05).

## 4. Discussion

The morphology of TMJ structures can be influenced by various factors, including tooth loss, tooth wear, premature contacts, parafunctional habits, unilateral crossbites, and dentoskeletal asymmetries [14]. TMJ morphology varies among individuals, and differences in functional loading have been suggested to be associated with variations in its shape. This phenomenon is based on the close relationship between form and function, supporting the assumed differences in mandibular condyle and glenoid fossa morphology among individuals with different types of malocclusion [15]. In this study, which evaluated the morphometric characteristics of the TMJ using CBCT images, significant differences were observed in the AEH and AEI, based on the occlusal relationship of the dental arches. Additionally, the AEI and GFD showed significant variations by gender. The AEI, along with the GFW and GFD, varied between sides. Furthermore, no correlation was found between age and the study variables.

The articular eminence, composed of thick and dense bone that adapts to mechanical forces and loads, is a crucial structure in TMJ biomechanics [16]. Studies have reported that morphological changes in the structure of the articular eminence may occur with advancing age, potentially leading to its flattening over time [17,18]. Studies evaluating gender differences in terms of AEH have reported higher mean AEH values in males compared to those in females [16,18,19]. Studies have emphasized that there is no significant difference in AEH between sides [20]. In this study, no significant difference was observed between sides in terms of AEH (*p* > 0.05), with mean measurements of 6.58 ± 1.21 mm on the right side and 6.64 ± 1.24 mm on the left side. The mean values observed in this study align with those reported in earlier research investigating the association between facial profile, malocclusion, TMJ dysfunctions, and AEH [13,21,22]. Our findings indicate that although AEH decreased with age on both sides, no significant correlation was found (*p* > 0.05), and the mean AEH was higher in males compared to in females, although the difference was not statistically significant (*p* > 0.05). In this study, articular eminence height was highest in the Angle Cl II group, followed by in the Cl III group, and lowest in the Cl I group. A statistically significant difference was observed only between the Angle Cl II and Cl I groups (*p* < 0.05), whereas no significant differences were found between the other study group comparisons (*p* > 0.05). A study that evaluated the height of the articular eminence, according to sagittal skeletal relationship [23], reported a significant difference between the Cl I and Cl III groups. García-Díaz et al. [24] did not find a significant difference in mean AEH between skeletal Cl I and Cl II groups. Although the mean AEH values in our study are generally consistent with the findings in the literature [23,24], variations in results may be attributed to differences in methodological approaches, such as classification of occlusion and the statistical methods employed. In the present study, the groups were defined according to Angle’s dental classification rather than via cephalometric skeletal classification. Therefore, dental occlusal relationships may not always fully correspond to the underlying craniofacial skeletal pattern, which should be considered when comparing our findings with studies based on skeletal Class I, Class II, and Class III patterns.

The morphology of the articular eminence shows individual variations, featuring different inclinations and generally exhibiting a convex shape [25]. In this study, two methods were used to evaluate the AEI: the best-fit line and top-roof line methods. The best-fit line method evaluates the inclination of the posterior surface of the articular eminence, whereas the top-roof line method assesses the relationship between the highest point of the articular eminence and the deepest point of the glenoid fossa [26]. Studies in the literature using both methods have consistently reported higher mean AEI values in males compared to those in females [16,18,27,28]. Similarly, in our study, the AEI was found to be significantly higher in males than in females using both methods (*p* < 0.05). In this study, no significant relationship was detected between AEI and age using either method (*p* > 0.05), which is consistent with the findings reported by Sa et al. [29] and Kim et al. [28]. In addition, our study revealed that AEI values on the left side were significantly higher than those on the right side, according to both the best-fit line and top-roof line methods (*p* < 0.05). In the literature, side-related differences in AEI have been associated with variations in functional loading, occlusal support, and adaptive remodeling of TMJ structures [28,30]. Factors affecting the TMJ can also influence the articular eminence and its inclination [31]. In the literature, it has been reported that individuals with Cl II division 2 malocclusion have higher AEI values compared to those with Cl I and Cl II division 1 malocclusions [13]. In contrast, Moscagiuri et al. [25] reported no significant differences between skeletal Cl I, Cl II, and Cl III groups on either side. In this study, a significant difference was observed among the study groups, with AEI values in individuals in the Angle Cl II group being significantly higher than those in the Cl I and Cl III groups using both methods (*p* < 0.05). This finding indicates that AEI may represent a sensitive morphometric parameter for detecting differences in osseous TMJ morphology among dental occlusal groups. Because the inclination of the articular eminence is closely related to the pathway of condylar translation, variations in AEI may have clinical relevance in CBCT-based TMJ evaluation, particularly in patients with Angle Class II occlusal relationships.

The size, position, and shape of the glenoid fossa can influence the shape, growth, and position of the mandibular condyle [32]. In this study, no significant difference was found in the mean GFW between genders (*p* > 0.05), which is consistent with the findings of Fan et al. [13]. Regarding the influence of age, Li et al. [33] observed an increase in GFW with age but reported no significant differences among age groups. Similarly, in this study, there was no significant relationship between age and GFW (*p* > 0.05). Alhammadi et al. [34] identified significant differences in GFW among individuals with Cl I, II, and III malocclusions, reporting the highest mean values in the Cl III group. In this study, the mean GFW was also wider in individuals in the Cl III group compared to those in the Cl I and Cl II groups; however, study group differences were not statistically significant (*p* > 0.05). Therefore, this observation was considered only as a descriptive finding and was not regarded as a significant group-related difference in glenoid fossa width.

The morphology of the glenoid fossa exhibits significant differences based on age and gender [35]. In this study, the mean GFD was determined to be significantly higher in males than in females (*p* < 0.05). Similarly, Ayyıldız et al. [36] reported significantly higher mean GFD values in males than in females. Conversely, the present study found no statistically significant relationship between the mean GFD and age (*p* > 0.05). However, Acuna et al. [37] found that the GFD decreased with age in cadavers aged between 20 and 85 years and reported a significant difference in mean GFD across age groups. These differences may be attributed to the inclusion of cadaveric samples in the study by Acuna et al. [37], as well as to the effects of advanced age and edentulism on the morphology of the glenoid fossa. In a study by Vitral et al. [38], which investigated individuals with Cl II division 1 subdivision malocclusions, no significant difference in mean GFD between the sides. Khademi et al. [39] and Fan et al. [13] found no significant difference in mean GFD among individuals with Cl I, Cl II, and Cl III malocclusions. The present study did not identify any statistically significant differences in mean GFD values among the groups (*p* > 0.05) [13,38,39]. Taken together, these findings suggest that GFD alone may have limited discriminative value in differentiating TMJ morphology among different occlusal relationships.

In the literature, ICA measurements have primarily focused on topics such as TMJ disorders and morphometric analyses, considering demographic and anatomical variables [36,40]. Generally, the ICA has been found to be lower in individuals with TMJ disorders and in patients with condylar resorption [40,41]. While some studies have emphasized the effect of gender [36], the influence of age has generally been found to be insignificant [42]. Nunez-Villaveiran et al. [42] measured the ICA as 135.64° on CT scans and 140.42° on dry skulls. Consistent with the literature, the present study found a mean ICA value of 138.45 ± 9.02°, and no statistically significant correlation was observed between age and ICA (*p* > 0.05). However, contrary to some findings reported in the literature, no statistically significant differences were observed between genders or among the study groups in the present study (*p* > 0.05). Fan et al. [13] reported that ICA varied among Cl I, Cl II division 1, and division 2 groups. This difference may be explained by variations in sample selection and occlusal classification, as Fan et al. [13] evaluated only skeletal Class I subjects and applied a more detailed dental subdivision of malocclusion, which may have resulted in clearer differentiation of angular measurements between groups.

In this study, the mean CA was measured as 69.53° and 69.81° on the right and left sides, respectively, with no significant differences observed between sides, genders, or groups (*p* > 0.05), and no association with age was found (*p* > 0.05). The findings are generally consistent with the results of other studies that have evaluated CA in different facial patterns, skeletal classifications, and dental arch structures [7,43,44,45]. Fan et al. [13] reported differences in mean CA among malocclusion groups, with the highest mean observed in individuals with Cl I occlusion (73.94°). Although there were no significant differences among the groups in the present study, similarly, the highest mean CA was observed in the Cl I group (70.57°).

This study has some limitations. Its retrospective, database-derived design restricted the ability to assess additional clinical characteristics such as bruxism, chewing side preference, and parafunctional habits. Patient anamnesis data were obtained exclusively through the clinical records, which may have limited the comprehensiveness of the available information. Furthermore, another limitation of the present study is that the occlusal relationship was evaluated based on Angle’s classification of the dental arches rather than on cephalometric skeletal classification. The relatively small number of patients in the Angle Class III group and the predominance of female patients in the sample should also be considered among the limitations of the present study. Additionally, the inclusion criteria of this study, which required patients to be fully dentate, resulted in a study population predominantly composed of younger individuals.

## 5. Conclusions

In conclusion, the occlusal relationship between the maxillary and mandibular dental arches was found to be associated with specific morphological characteristics of the TMJ, particularly the articular eminence height and inclination, while no significant association was observed for other TMJ parameters. In addition, gender and side-related differences were identified in specific TMJ parameters. These findings underscore the importance of considering such variations in both the physiological assessment and pathological evaluation of TMJ anatomy.

## Figures and Tables

**Table 1:** Descriptive statistics of the articular eminence height.

Gender	Group	Side						Total		
		Right			Left					
		*n*	Mean	SD	*n*	Mean	SD	*n*	Mean	SD
Female	Angle Class I	36	6.17	1.22	36	6.22	1.26	72	6.20	1.23
	Angle Class II	54	6.83	1.01	54	6.90	1.01	108	6.86	1.01
	Angle Class III	6	5.81	0.67	6	6.00	0.64	12	5.90	0.63
	Total	96	6.52	1.13	96	6.59	1.14	192	6.55	1.13
Male	Angle Class I	14	6.48	1.20	14	6.43	1.26	28	6.45	1.20
	Angle Class II	11	7.28	1.39	11	7.19	1.26	22	7.23	1.29
	Angle Class III	10	6.61	1.65	10	6.77	1.96	20	6.69	1.76
	Total	35	6.77	1.40	35	6.77	1.48	70	6.77	1.43
Total	Angle Class I	50	6.26	1.21	50	6.28	1.25	100	6.27 ^b^	1.22
	Angle Class II	65	6.90	1.09	65	6.95	1.05	130	6.93 ^a^	1.07
	Angle Class III	16	6.31	1.39	16	6.48	1.61	32	6.40 ^ab^	1.48
	Total	131	6.58	1.21	131	6.64	1.24	262	6.61	1.22
*p* *	Side: *p* = 0.387, ηp^2^ = 0.006; Gender: *p* = 0.078, ηp^2^ = 0.025; Group: *p* = 0.014, ηp^2^ = 0.066; Side × Gender Int.: *p* = 0.466, ηp^2^ = 0.004; Side × Group Int.: *p* = 0.535, ηp^2^ = 0.010; Gender × Group Int.: *p* = 0.746, ηp^2^ = 0.005; Side × Gender × Group Int.: *p* = 0.933, ηp^2^ = 0.001.									

SD: standard deviation; Int: interaction; ηp^2^: partial eta squared; *: three-way repeated-measure ANOVA. According to the Bonferroni test, the difference between means with no common letter is significant (*p* < 0.05).

**Table 2:** Descriptive statistics of the articular eminence inclination measured using the best-fit line method.

Gender	Group	Side						Total		
		Right			Left					
		*n*	Mean	SD	*n*	Mean	SD	*n*	Mean	SD
Female	Angle Class I	36	47.05	5.75	36	48.23	5.85	72	47.64	5.79
	Angle Class II	54	50.50	5.69	54	51.90	4.63	108	51.20	5.21
	Angle Class III	6	44.02	3.53	6	46.51	3.98	12	45.26	3.81
	Total	96	48.80	5.93	96	50.18	5.42	192	49.49	5.71
Male	Angle Class I	14	49.54	4.88	14	52.18	6.48	28	50.86	5.79
	Angle Class II	11	54.12	7.74	11	54.73	7.70	22	54.43	7.54
	Angle Class III	10	47.73	7.60	10	49.31	10.05	20	48.52	8.71
	Total	35	50.46	6.99	35	52.16	8.05	70	51.31	7.53
Total	Angle Class I	50	47.75	5.58	50	49.33	6.23	100	48.54 ^b^	5.94
	Angle Class II	65	51.11	6.17	65	52.37	5.31	130	51.74 ^a^	5.77
	Angle Class III	16	46.34	6.50	16	48.26	8.24	32	47.30 ^b^	7.36
	Total	131	49.24	6.24	131	50.71	6.26	262	49.98	6.28
*p* *	Side: *p* = 0.001, ηp^2^ = 0.084; Gender: *p* = 0.012, ηp^2^ = 0.049; Group: *p* = 0.001, ηp^2^ = 0.104; Side × Gender Int.: *p* = 0.932, ηp^2^ = 0.000; Side × Group Int.: *p* = 0.578, ηp^2^ = 0.009; Gender × Group Int.: *p* = 0.999, ηp^2^ = 0.000; Side × Gender × Group Int.: *p* = 0.437, ηp^2^ = 0.013.									

SD: standard deviation; Int: interaction; ηp^2^: partial eta squared; *: three-way repeated-measure ANOVA. According to the Bonferroni test, the difference between means with no common letter is significant (*p* < 0.05).

**Table 3:** Descriptive statistics of the articular eminence inclination measured using the top-roof line method.

Gender	Group	Side						Total		
		Right			Left					
		*n*	Mean	SD	*n*	Mean	SD	*n*	Mean	SD
Female	Angle Class I	36	34.90	4.61	36	35.54	4.19	72	35.22	4.39
	Angle Class II	54	37.01	4.87	54	38.41	4.60	108	37.71	4.77
	Angle Class III	6	30.85	2.34	6	34.00	4.08	12	32.43	3.57
	Total	96	35.83	4.91	96	37.06	4.65	192	36.45	4.81
Male	Angle Class I	14	36.83	3.81	14	37.97	4.70	28	37.40	4.24
	Angle Class II	11	40.96	6.85	11	41.10	5.83	22	41.03	6.21
	Angle Class III	10	34.24	5.14	10	35.86	6.74	20	35.05	5.89
	Total	35	37.38	5.79	35	38.35	5.90	70	37.87	5.82
Total	Angle Class I	50	35.44	4.45	50	36.22	4.43	100	35.83 ^b^	4.43
	Angle Class II	65	37.68	5.41	65	38.86	4.88	130	38.27 ^a^	5.17
	Angle Class III	16	32.97	4.53	16	35.16	5.81	32	34.07 ^b^	5.24
	Total	131	36.25	5.18	131	37.40	5.02	262	36.83	5.13
*p* *	Side: *p* = 0.001, ηp^2^ = 0.086; Gender: *p* = 0.010, ηp^2^ = 0.052; Group: *p* < 0.001, ηp^2^ = 0.132; Side × Gender Int.: *p* = 0.338, ηp^2^ = 0.007; Side × Group Int.: *p* = 0.280, ηp^2^ = 0.020; Gender × Group Int.: *p* = 0.856, ηp^2^ = 0.002; Side × Gender × Group Int.: *p* = 0.443, ηp^2^ = 0.013.									

SD: standard deviation; Int: interaction; ηp^2^: partial eta squared; *: three-way repeated-measure ANOVA. According to the Bonferroni test, the difference between means with no common letter is significant (*p* < 0.05).

**Table 4:** Descriptive statistics of the glenoid fossa width.

Gender	Group	Side						Total		
		Right			Left					
		*n*	Mean	SD	*n*	Mean	SD	*n*	Mean	SD
Female	Angle Class I	36	18.10	1.15	36	17.87	1.21	72	17.99	1.18
	Angle Class II	54	18.16	1.05	54	17.88	1.25	108	18.02	1.16
	Angle Class III	6	18.45	1.36	6	17.88	2.01	12	18.17	1.66
	Total	96	18.16	1.01	96	17.88	1.27	192	18.02	1.19
Male	Angle Class I	14	18.45	1.09	14	18.09	1.39	28	18.27	1.24
	Angle Class II	11	18.12	1.19	11	18.15	1.47	22	18.14	1.31
	Angle Class III	10	18.57	0.72	10	18.49	1.23	20	18.53	0.98
	Total	35	18.38	1.02	35	18.22	1.34	70	18.30	1.19
Total	Angle Class I	50	18.20	1.13	50	17.93	1.25	100	18.07	1.19
	Angle Class II	65	18.16	1.06	65	17.98	1.28	130	18.04	1.18
	Angle Class III	16	18.53	0.97	16	18.26	1.53	32	18.39	1.27
	Total	131	18.22	1.08	131	17.97	1.30	262	18.09	1.20
*p* *	Side: *p* = 0.029, ηp^2^ = 0.038; Gender: *p* = 0.316, ηp^2^ = 0.008; Group: *p* = 0.721, ηp^2^ = 0.005; Side × Gender Int.: *p* = 0.332, ηp^2^ = 0.008; Side × Group Int.: *p* = 0.698, ηp^2^ = 0.006; Gender × Group Int.: *p* = 0.912, ηp^2^ = 0.001; Side × Gender × Group Int.: *p* = 0.482, ηp^2^ = 0.012.									

SD: standard deviation; Int: interaction; ηp^2^: partial eta squared; *: three-way repeated-measure ANOVA.

**Table 5:** Descriptive statistics of the glenoid fossa depth.

Gender	Group	Side						Total		
		Right			Left					
		*n*	Mean	SD	*n*	Mean	SD	*n*	Mean	SD
Female	Angle Class I	36	6.43	0.88	36	6.32	0.84	72	6.38	0.86
	Angle Class II	54	6.61	0.79	54	6.43	0.85	108	6.52	0.82
	Angle Class III	6	7.19	1.20	6	6.71	1.35	12	6.95	1.24
	Total	96	6.58	0.86	96	6.41	0.88	192	6.49	0.87
Male	Angle Class I	14	7.39	1.30	14	6.98	1.25	28	7.18	1.27
	Angle Class II	11	6.77	0.59	11	6.78	0.82	22	6.78	0.69
	Angle Class III	10	7.67	0.78	10	7.57	0.88	20	7.62	0.81
	Total	35	7.27	1.02	35	7.09	1.05	70	7.18	1.03
Total	Angle Class I	50	6.70	1.09	50	6.51	1.01	100	6.60	1.05
	Angle Class II	65	6.64	0.75	65	6.49	0.85	130	6.56	0.80
	Angle Class III	16	7.49	0.95	16	7.25	1.12	32	7.37	1.03
	Total	131	6.77	0.95	131	6.59	0.97	262	6.68	0.96
*p* *	Side: *p* = 0.004, ηp^2^ = 0.065; Gender: *p* = 0.004, ηp^2^ = 0.066; Group: *p* = 0.052, ηp^2^ = 0.046; Side × Gender Int.: *p* = 0.510, ηp^2^ = 0.003; Side × Group Int.: *p* = 0.367, ηp^2^ = 0.016; Gender × Group Int.: *p* = 0.359, ηp^2^ = 0.016; Side × Gender × Group Int.: *p* = 0.104, ηp^2^ = 0.036.									

SD: standard deviation; Int: interaction; ηp^2^: partial eta squared; *: three-way repeated-measure ANOVA.

**Table 6:** Descriptive statistics of the intercondylar angle.

Gender	Group	*n*	Mean	SD
Female	Angle Class I	36	140.43	9.06
	Angle Class II	54	138.31	7.92
	Angle Class III	6	132.58	11.27
	Total	96	138.75	8.69
Male	Angle Class I	14	140.07	11.76
	Angle Class II	11	135.01	7.82
	Angle Class III	10	137.15	9.44
	Total	35	137.64	9.96
Total	Angle Class I	50	140.33	9.77
	Angle Class II	65	137.75	7.94
	Angle Class III	16	135.43	10.05
	Total	131	138.45	9.02
*p* *	Gender: *p* = 0.884, ηp^2^ = 0.000; Group: *p* = 0.078, ηp^2^ = 0.040; Gender × Group Int.: *p* = 0.357, ηp^2^ = 0.016.			

SD: standard deviation; Int: interaction; ηp^2^: partial eta squared; *: two-way ANOVA.

**Table 7:** Descriptive statistics of the condylar angle.

Gender	Group	Side						Total		
		Right			Left					
		*n*	Mean	SD	*n*	Mean	SD	*n*	Mean	SD
Female	Angle Class I	36	70.41	4.31	36	70.93	4.37	72	70.67	4.32
	Angle Class II	54	69.72	3.69	54	69.74	3.80	108	69.73	3.73
	Angle Class III	6	67.55	5.88	6	68.05	5.16	12	67.80	5.28
	Total	96	69.84	4.09	96	70.08	4.13	192	69.96	4.10
Male	Angle Class I	14	70.37	4.48	14	70.28	5.56	28	70.33	4.95
	Angle Class II	11	67.40	4.76	11	67.80	4.09	22	67.60	4.33
	Angle Class III	10	67.75	4.75	10	68.77	4.32	20	68.26	4.45
	Total	35	68.69	4.72	35	69.07	4.78	70	68.88	4.72
Total	Angle Class I	50	70.40	4.31	50	70.74	4.68	100	70.57	4.48
	Angle Class II	65	69.33	3.95	65	69.42	3.89	130	69.37	3.90
	Angle Class III	16	67.67	5.01	16	68.50	4.49	32	68.08	4.70
	Total	131	69.53	4.28	131	69.81	4.32	262	69.67	4.29
*p* *	Side: *p* = 0.145, ηp^2^ = 0.017; Gender: *p* = 0.475, ηp^2^ = 0.004; Group: *p* = 0.060, ηp^2^ = 0.044; Side × Gender Int.: *p* = 0.861, ηp^2^ = 0.000; Side × Group Int.: *p* = 0.708, ηp^2^ = 0.006; Gender × Group Int.: *p* = 0.492, ηp^2^ = 0.011; Side × Gender × Group Int.: *p* = 0.579, ηp^2^ = 0.009.									

SD: standard deviation; Int: interaction; ηp^2^: partial eta squared; *: three-way repeated-measure ANOVA.

**Table 8:** Model-based effect-size estimates and 95% confidence intervals for statistically significant comparisons.

Outcome	Comparison	Mean Difference (95% CI)	Cohen’s d	*p*
Articular eminence height	Angle Class II—Angle Class I	0.72 mm (0.09 to 1.36 mm)	0.628	0.021
Articular eminence inclination, best-fit line	Angle Class II—Angle Class I	3.56° (0.48 to 6.64°)	0.642	0.017
Articular eminence inclination, best-fit line	Angle Class II—Angle Class III	5.92° (1.79 to 10.05°)	1.066	0.002
Articular eminence inclination, best-fit line	Left—Right	1.65° (0.69 to 2.61°)	0.390	<0.001
Articular eminence inclination, best-fit line	Male—Female	3.23° (0.71 to 5.76°)	0.583	0.012
Articular eminence inclination, top-roof line	Angle Class II—Angle Class I	3.06° (0.57 to 5.55°)	0.681	0.010
Articular eminence inclination, top-roof line	Angle Class II—Angle Class III	5.63° (2.29 to 8.97°)	1.254	0.001
Articular eminence inclination, top-roof line	Left—Right	1.35° (0.57 to 2.13°)	0.393	0.001
Articular eminence inclination, top-roof line	Male—Female	2.71° (0.66 to 4.75°)	0.603	0.010
Glenoid fossa width	Right—Left	0.25 mm (0.03 to 0.48 mm)	0.254	*p* = 0.029
Glenoid fossa depth	Male—Female	0.58 mm (0.19 to 0.96 mm)	0.681	*p* = 0.004
Glenoid fossa depth	Right—Left	0.21 mm (0.07 to 0.35 mm)	0.340	*p* = 0.004

CI: confidence interval. Mean differences and confidence intervals were obtained from the factorial/repeated-measures ANOVA model. Group-comparison *p*-values are Bonferroni-adjusted. Cohen’s d values were calculated using the model residual standard deviation for between-subject contrasts and the residual standard deviation of the side-difference model for side contrasts.

**Table 9:** Correlation coefficients between age and study variables.

	r	*p*
Intercondylar Angle	−0.103	0.240
Right Condylar Angle	−0.086	0.331
Left Condylar Angle	−0.121	0.168
Right Articular Eminence Height	−0.038	0.666
Left Articular Eminence Height	−0.071	0.422
Right Articular Eminence Inclination, Best-Fit Line Method	−0.017	0.845
Left Articular Eminence Inclination, Best-Fit Line Method	−0.015	0.868
Right Articular Eminence Inclination, Top-Roof Line Method	−0.016	0.856
Left Articular Eminence Inclination, Top-Roof Line Method	−0.018	0.841
Right Glenoid Fossa Width	−0.057	0.520
Left Glenoid Fossa Width	−0.017	0.848
Right Glenoid Fossa Depth	−0.091	0.301
Left Glenoid Fossa Depth	−0.071	0.421

r: Spearman’s rank correlation coefficient.

## Data Availability

The datasets used and/or analyzed during the current study are available from the corresponding author on reasonable request.
